# Comparison of chemical and microbiological changes during the aerobic composting and vermicomposting of green waste

**DOI:** 10.1371/journal.pone.0207494

**Published:** 2018-11-26

**Authors:** Linlin Cai, Xiaoqiang Gong, Xiangyang Sun, Suyan Li, Xin Yu

**Affiliations:** College of Forestry, Beijing Forestry University, Beijing, P.R. China; Universita degli Studi di Milano-Bicocca, ITALY

## Abstract

This research was conducted to compare chemical and microbiological properties during aerobic composting (AC) and vermicomposting (VC) of green waste. Relative to AC, VC significantly decreased the pH and lignin and cellulose contents, and significantly increased the electrical conductivity and total N and available P contents. For AC, *BIrii41_norank* (order Myxococcales) was the major bacterial genus at 30 d and again became dominant genus from 90–150 d, with relative abundances of 2.88% and 4.77–5.19%, respectively; at 45 d and 60 d, the dominant bacterial genus was *Nitrosomonadaceae_uncultured* (order Nitrosomonadales) with relative abundances of 2.83–7.17%. For VC, the dominant bacterial genus was *BIrii41_norank* (except at 45 d), which accounted for 2.11–7.96% of the total reads. The dominant fungal class was Sordariomycetes in AC (relative abundances 39.2–80.6%) and VC (relative abundances 42.1–69.5%). The abundances of microbial taxa and therefore the bacterial and fungal community structures differed between VC and AC. The quality of the green waste compost product was higher with VC than with AC. These results will also help to achieve further composting technology breakthroughs in reducing the composting time and improving compost quality.

## Introduction

Given the need for environmental sustainability, the increasing cost of urban green waste treatment, and the cost of importing horticultural substrates, increasing attention is being paid to transforming green waste into useful horticultural products via composting in China and other countries. The rapid development of urban green spaces has resulted in the generation of substantial quantities of green waste in China. Beijing City alone produces 6 million tons of green waste annually [1]. Garden waste disposal commonly involves large-scale incineration or deposition in landfills, but both are environmentally harmful and fail to make use of a potentially valuable resource [2].

According to the different ways of recycling organic solid waste, composting can be divided into three types: anaerobic fermentation, aerobic composting, and vermicomposting. As noted earlier, green waste can be converted into useful products by composting. In aerobic composting, organic waste are converted into compost by microorganisms under aerobic conditions. The types and numbers of microorganisms can affect the composting process and the physical and chemical properties of the product. Several methods have been studied to improve aerobic compost quality and fermentation performance, such as the addition of microbial agents and specific-purpose regulators. Gong et al. [3] observed that compost additives containing *Trametes versicolor* and *Phanerochaete chrysosporium* increased the lignin and cellulose degradation rates, reduced the time required for the compost to attain maturity, increased the nutrient content, and eliminated phytotoxicity in the final product. Addition of the fungi *Trichoderma viride*, *Aspergillus niger*, and *Aspergillus flavus* to the composting materials increased the mineralization of C and N and resulted in a stable and non-phytotoxic compost [4]. Organic additives like ​cow manure and coffee grounds contain microorganisms and also can enhance the activity of the microorganisms already in green waste during aerobic composting; these additives can increase the concentration of low-molecular-weight compounds such as glucose, methoxyphenol, and superoxide anion groups; increase the activities of dehydrogenases, cellulases, and phosphatases; and improve particle size distribution so as to maintain plant-available water-holding capacity and aeration [5]. However studies on the dynamic changes of the microbial community during aerobic composting of greening waste are scarce.

Vermicomposting is a rapid, simple, easy-to-control, energy-saving, and cost-effective composting process that can produce a valuable product [6, 7]. It is especially suitable for the disposal of scattered, urban green waste at the location where the green waste is produced. Previous studies have demonstrated that vermicomposting can transform green waste into useful compost [8, 9, 10]. Vermicomposting is the process of biochemical oxidation and transformation of organic matter through the interaction of earthworms and microorganisms at room temperature and under aerobic conditions [2]. During vermicomposting, organic matter is decomposed by the activities of earthworms and of the microorganisms in the earthworms and in the original substrate [11], fungi are especially important for the degradation of lignocellulose and other degradation-resistant substances that are abundant in green waste [12]. It follows that studying the effects of earthworms on the activity, quantity, and community structure of microorganism can increase our understanding of vermicomposting. Huang et al. [13] who studied the vermicomposting of vegetable waste and cow dung, found that microbial numbers are reduced but microbial diversity is increased by passage through the earthworm intestinal transit. Although previous studies documented the microbial community in some final vermicompost products, changes in microbial communities during vermicomposting of green waste still require further investigation.

The purpose of this study was to compare the effects of aerobic composting and vermicomposting of green waste on bacterial and fungal communities throughout both processes and to compare the effects of the two composting methods on the quality of the final product.

## Materials and methods

### Ethics statement

The experiment was carried out in our scientific research greenhouse which is owned by our institute, therefore, no specific permissions were required for these locations/activities. We also confirm that the studies did not involve endangered or protected species.

### Experimental design

An experiment was conducted at the nursery of Beijing Forestry University Forestry Technology Co., Ltd. The green waste (consisting mainly of trimmings and litter of *Robinia pseudoacacia* Linn., *Cotinus coggygria* Scop., and *Ilex chinensis* Sims) was collected from the Beijing Botanical Garden, Haidian District, Beijing. Before the experiment began, green waste was chopped into 5-cm-diameter pieces and its moisture content was increased to 60–70% (w/w). The main chemical properties of the green waste are listed in Table 1. For both aerobic and vermicomposting, 40 kg of substrate was placed in uncovered plastic bins (0.6 m wide ⊆ 0.8 m long ⊆ 0.65 m high). The bottom of each bin had 20 holes (10 mm diameter) for discharge of leachate. A plastic mesh with 1-mm openings was used to cover the bottom of each bin to prevent earthworm escape in vercomposting. Each of the two treatments was represented by three replicate bins.

**Table 1:** Chemical properties of the green waste, the aerobic compost product, and the vermicompost product.

	Chemical property							
compost product	pH	EC (ds m^-1^)	TOC (g kg^-1^)	TKN (g kg^-1^)	Available P (g kg^-1^)	C/N ratio	Cellulose (%)	Lignin (%)
Green waste	6.5±0.1 c	0.5±0.0 c	432.3±6.9 a	12.6±0.3 c	0.5±0.1 c	34.5±1.2 c	55.02±0.3 a	32.8±1.9 a
Aerobic compost	8.2±0.0 a	1.6±0.1 b	325.9±2.3 b	22.9±0.3 b	0.8±0.1 b	14.2±0.3 b	24.0±0.8 b	17.2±0.4 b
Vermicompost	7.7±0.0 b	1.9±0.0 a	295.1±4.4 c	25.6±0.5 a	1.1±0.1 a	11.5±0.1 a	19.6±0.4 c	13.2±0.4 c

Means in a column followed by different letters are significantly different at P<0.05 according to the LSD test.

For aerobic composting, the materials were manually removed from each bin weekly, and the material was repeatedly inverted with a shovel until uniform to ensure adequate aeration and to enhance decomposition, and was then returned to the bin.

For vermicomposting, this same process was performed weeks, which was before earthworms were added. At 15 d, adults of the earthworm *Eisenia fetida* were added at a rate of 20/kg of dry material. The earthworms had been reared in laboratory cultures containing cow dung and had an average (± SE) weight of 419 ± 38 mg. Thereafter, the materials in the vermicomposting bins were not disturbed except sampling. The substrate in all bins was periodically sprinkled with sterile water to maintain moisture at 60–70%.

Samples were collected at 30, 45, 60, 90, 120, and 150 d. At each time, an equivalent mass was collected at three different locations for each of three depths in each replicate. The nine samples were mixed and then divided into two subsamples (earthworms and cocoons were manually separated and returned to the bins). One subsample was stored at -20°C for DNA analysis, and the other was air-dried and ground for chemical analysis.

### Chemical analysis

pH and electrical conductivity (EC) were measured using a MP521 PH/EC meter (Shanghai, China) in aqueous suspensions with a solid to deionized water ratio of 1:10 (w/v). Total Kjeldahl nitrogen (TKN) content was determined with an automated Kjeldahl nitrogen analyzer (KDY-9830; Beijing Tongrunyuan Electromechanical Technology Co., Ltd., Beijing, China). The available phosphorus (P) content was determined by Anti-Mo-Sb spectrophotometry with a spectrophotometer (UV-120-02; Shimadzu Scientific Instruments, Kyoto, Japan) as described by Fu et al. [14]. The total organic carbon (TOC) content was measured with a Shimadzu TOC-Vcp total organic carbon analyzer (Kyoto, Japan). The cellulose content was analyzed by the HNO_3_-ethanol method [15]. The lignin content was determined with the 72% (v/v) H_2_SO_4_ method as previously described by Liu [15].

### DNA extraction and PCR amplification

Samples of aerobic compost and vermicompost were collected on days 30, 45, 60, 90, 120, and 150 and stored at -20°C for analyze the microbial community composition during the compost process. The microbial DNA of aerobic compost and vermicompost samples extraction using the Mo Bio Power Soil DNA Isolation Kit (Mo Bio Laboratory, Carlsbad, CA, USA) according to the manufacturer's protocol. A Nano Drop 2000 spectrophotometer (Thermo Scientific, Inc., Waltham, MA, USA) was used to measure the concentration and purity of the extracted DNA.

The primer pair of 806R (5'- GGA CTA CHV GGG TWT CTA AT-3') and 338F (5'-ACT CCT ACG GGA GGC AGC AG-3') was used to amplify 16S rDNA gene of bacteria, with a PCR program as follows, initial denaturation at 95°C for 5 minutes, followed by 35 cycles of 30 s at 95°C, 30 s at 58°C, 40 s at 72°C, a final extension at 72°C for 10 min. The primer pair of SSU 1196R (5'-TCT GGA CCT GGT GAG TTT CC-3') and SSU 0817F (5'- TTA GCA TGG AAT AAT RRA ATA GGA-3') was used to amplify the 18S rDNA gene of fungi, and the PCR program included an For fungi, the PCR program included an initial denaturation at 94°C for 5 min; followed by 35 cycles 95°C for 30 s, 55°C for 30 s, and 72°C for 40 s; and a final extension at 72°C for 10 min. PCR amplification was performed using a T100 Thermal Cycler (Bio-rad Laboratories, Inc, Hercules, USA). PCR amplification products were electrophoresed with 2% (w/v) agarose plus 0.4 μg/ml of Ethidium Bromide for 15–20 min to determine whether the they were correct.

The DNA samples were sequenced using the Illumina MiSeq PE300 platform at Shanghai Majorbio Biomedical Technology Co., Ltd. (Shanghai, China). At the time of quality control, any sequence containing mismatches and fuzzy readings (N) in the primers were removed. After removing the failed sequence, the total valid sequences of all 36 samples was 9253224. UCHIME was then used to screen these clean and non-contiguous sequences of chimeras. The silva database was employed for aligning the sequences (http://www.arb-silva.de/). Cluster operational taxonomic units (OTUs) was applied to Usearch with a similarity level of 97% (vsesion7.1 http://drive5.com/uparse/). Mothur was used to create Alpha diversity of samples that mainly contained the Chao richness estimator, Shannon index, and Good's coverage (version: 1.30.1, http://www.mothur.org/wiki/Classify.seqs). The ribosome database ‘project algorithm’ was used to classify the phylum and genera levels. Principal component analysis was performed at 3% dissimilar level.

### Statistical analysis

One-way analyses of variance (ANOVAs) were used to compare the chemical properties of the final composts. Mean values were separated by the least significant difference (LSD) test at the 5% level. All statistical analyses were performed using SPSS 21.0 software (Chicago, USA).

## Results and discussion

### Chemical properties of the green waste and the final compost products

Vermicomposting and aerobic composting of green waste resulted in decreases in TOC, C/N, lignin, and cellulose, and increases in pH, EC, TN, and available P in the final products. pH, TOC, C/N ratio, and cellulose and lignin percentages were lower in vermicompost than in aerobic compost. EC, TKN, and available P were higher in vermicompost than in aerobic compost. Similar findings were reported by Pratibha et al. [16]. The greater decrease of pH in vermicompost than in aerobic compost could have been caused by earthworm acceleration of organic matter mineralization, which results in the production of organic acids [17]. The increase in EC value, TN, and available P in vermicompost might also be due to accelerated degradation caused by earthworms. Suthar [18] suggested that the release of nitrogenous products by earthworms during vermicomposting can also increase the TN content of the final product.

The C/N ratio, which was lower in the vermicompost than in aerobic compost, has traditionally been considered to indicate compost maturity. According to Van Heerden et al. [19], a C/N ratio below 20 indicates that the compost is mature, and a ratio below 15 is preferred for composts used in agronomy. In the current study, both vermicompost and aerobic compost had a C/N ratio below 15.

The reductions in TOC, lignin, and cellulose were greater in the vermicompost than in the aerobic compost. The faster decomposition of green waste with vermicomposting than with aerobic process can be explained by the fragmentation of organic matter by earthworms, which increases the surface area of the organic matter and thereby increases the quantity of organic matter accessible to microorganisms and microbial enzymes [20].

### Analysis of microbial richness and diversity during aerobic composting and vermicomposting of green waste

The microbial richness and diversity of the two treatments during various stages of composting were calculated based on 3084408 sequencing reads that were randomly picked from each sample. Reads sharing 97% nucleotide sequence identity were grouped into operational taxonomic units, resulting in 2144 to 3099 OTUs (Table 2). OTUs and the Chao index were higher in vermicompost samples than in aerobic compost samples throughout the composting process. The Shannon diversity index was also higher in vermicompost samples than in aerobic compost samples throughout the composting process. The coverage value of all the samples was estimated to be > 99% and did not significantly differ between the two treatments. The results suggested that microbial richness and diversity were higher with vermicomposting than with aerobic composting. These observations are consistent with the results obtained by Vivas et al. [8], who reported that bacterial population size and diversity were enhanced during composting of olive-mill waste when *E*. *fetida* was present. Earthworms host millions of microorganisms in their guts and excrete them in their casts [21]. Moreover, earthworms enhance decomposition, and earthworm casts are rich in available, soluble nutrients that would enhance bacterial multiplication [22].

**Table 2:** Diversity statistics for the microbial community during aerobic composting (AC) and vermicomposting (VC) of green waste.

Compost time (d)		Reads	OTUs	Chao	Shannon	Coverage
30	AC	257034	2144	2368	8.19	0.99
	VC	257034	2749	3112	8.33	0.99
45	AC	257034	2271	2580	8.20	0.99
	VC	257034	2875	3198	7.95	0.99
60	AC	257034	2322	2599	7.50	0.99
	VC	257034	2894	3356	8.26	0.99
90	AC	257034	2510	2927	7.65	0.99
	VC	257034	2841	3275	8.31	0.99
120	AC	257034	2821	3181	9.08	0.99
	VC	257034	2999	3365	9.27	0.99
150	AC	257034	2875	3289	8.91	0.99
	VC	257034	3099	3466	8.88	0.99

**Fig 1:**
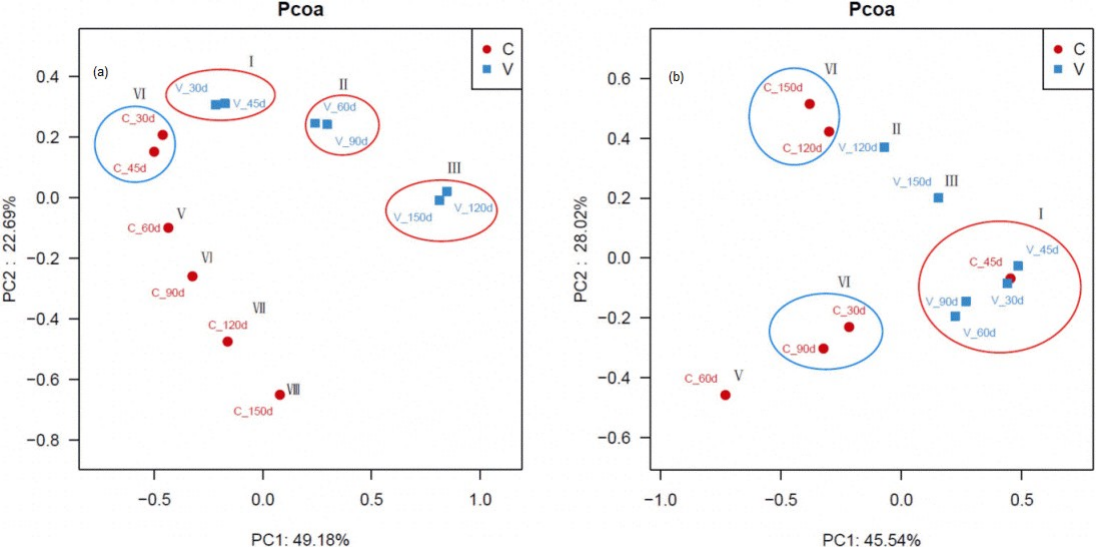
Principle component analysis (PCA) of a) bacterial community structure b) fungal community structure in vermicompost samples (blue symbols and labels) and aerobic compost samples (red symbols and labels). The sampling day is indicated by the numerical value in the label.

The variance in bacterial and fungal community structure during aerobic composting and vermicomposting was assessed by principle component analyses (Fig 1). For bacterial community structure, component 1 (PC1) and 2 (PC2) accounted for 49.2% and 22.7% of the total variance in OTUs, respectively (Fig 1A). All bacterial samples were divided into eight groups. The vermicompost samples from 30 d and 45 d were in Group I, which was identical to Group Ⅳ, and all of them had a similar bacterial community structure. This period is the thermophilic phase of aerobic composting and is dominated by thermophilic microorganisms [23], whereas vermicomposting is at the mesophilic stage in which earthworm plays a role in composting green waste after 2 weeks of thermophilic pretreatment, indicating that thermophilic bacteria can still survival under mesophilic conditions [24]. Group Ⅱ included the vermicompost samples from 60 d and 90 d, which corresponds to the mesophilic phase of vermicomposting, when bacterial abundance increases but bacterial diversity decreases (Table 2). The vermicompost samples collected at 120 d and 150 d were also similar, indicating that the bacterial community structure was relatively stable at the curing stage. The differences among aerobic compost samples taken at 60 d, 90 d, 120 d, and 150 d indicate that the bacterial community structure during the thermophilic phase differed from the structure before and after the thermophilic phase.

For fungal community structure, PC1 and PC2 explained 45.5% and 28.0% of the total variance in OTUs, respectively (Fig 1B). The fungal sequences were divided into six groups. Group Ⅰ, which was identical to Group IV, included the 30- to 90-d samples from vermicomposting and the 30-d sample from aerobic composting. This period mostly involved the mesophilic phase in vermicomposting, when mesophilic fungi play a dominant role. The fungal community structure in the vermicompost was clearly different at 120 d and 150 d than at earlier times. The fungal community structure in the aerobic compost was similar at 120 d and 150 d, indicating that the fungal community structure entered a relatively stable curing period after 90 d of aerobic composting.

**Fig 2:**
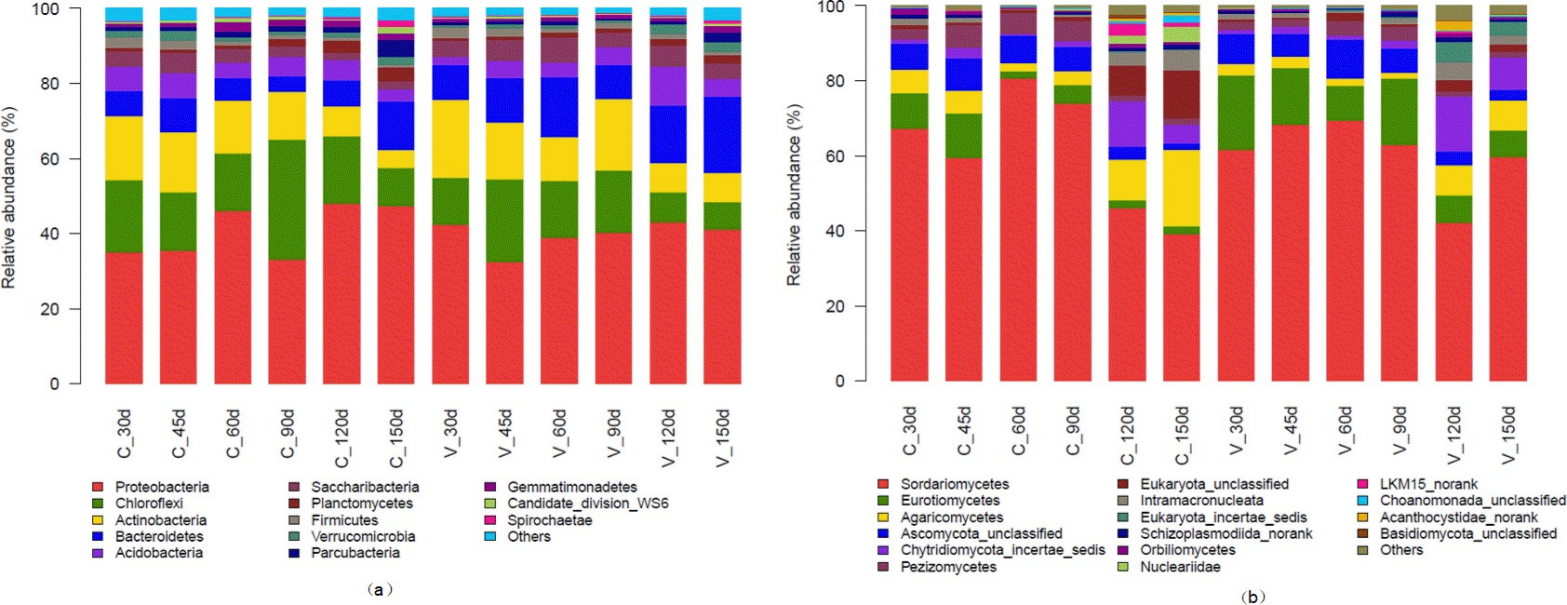
The relative abundance of bacterial phyla (a) and fungal and other phyla (b) at different times after initiation of aerobic composting and vermicomposting.

The relative abundances of the dominant bacterial and fungal phyla in the aerobic compost and vermicompost at different sample times are shown in Fig 2. The same phyla dominated in both kinds of compost, but the relative abundance of each phylum was different. The dominant bacterial phylum was *Proteobacteria*, followed by *Chloroflexi*, *Actinobacteria*, *Bacteroidetes*, *Acidobacteria*, and *Saccharibacteria*. These phyla accounted for 88.3–90.9% of the sequences in the aerobic compost and for 90.1–93.5% of the sequences in the vermicompost (excluding the sample from 150 d). The relative abundances of the following phyla were <1% in all samples: *Planctomycetes*, *Firmicutes*, *Verrucomicrobia*, *Parcubacteria*, *Gemmatimonadetes*, *Candidate_division_WS6*, and *Spirochaetae*.

The fungal phyla *Ascomycota*, *Basidiomycota*, *Chytridiomycota*, *Zygomycota*, and *Cryptomycota* were detected in all compost samples and accounted for 71.3–98.7% of the sequences in the aerobic compost and for 79.1–97.6% of the sequences in the vermicompost. The remaining phyla belonged to the kingdoms *Protozoa*, *Chromista*, and *Animalia*.

**Fig 3:**
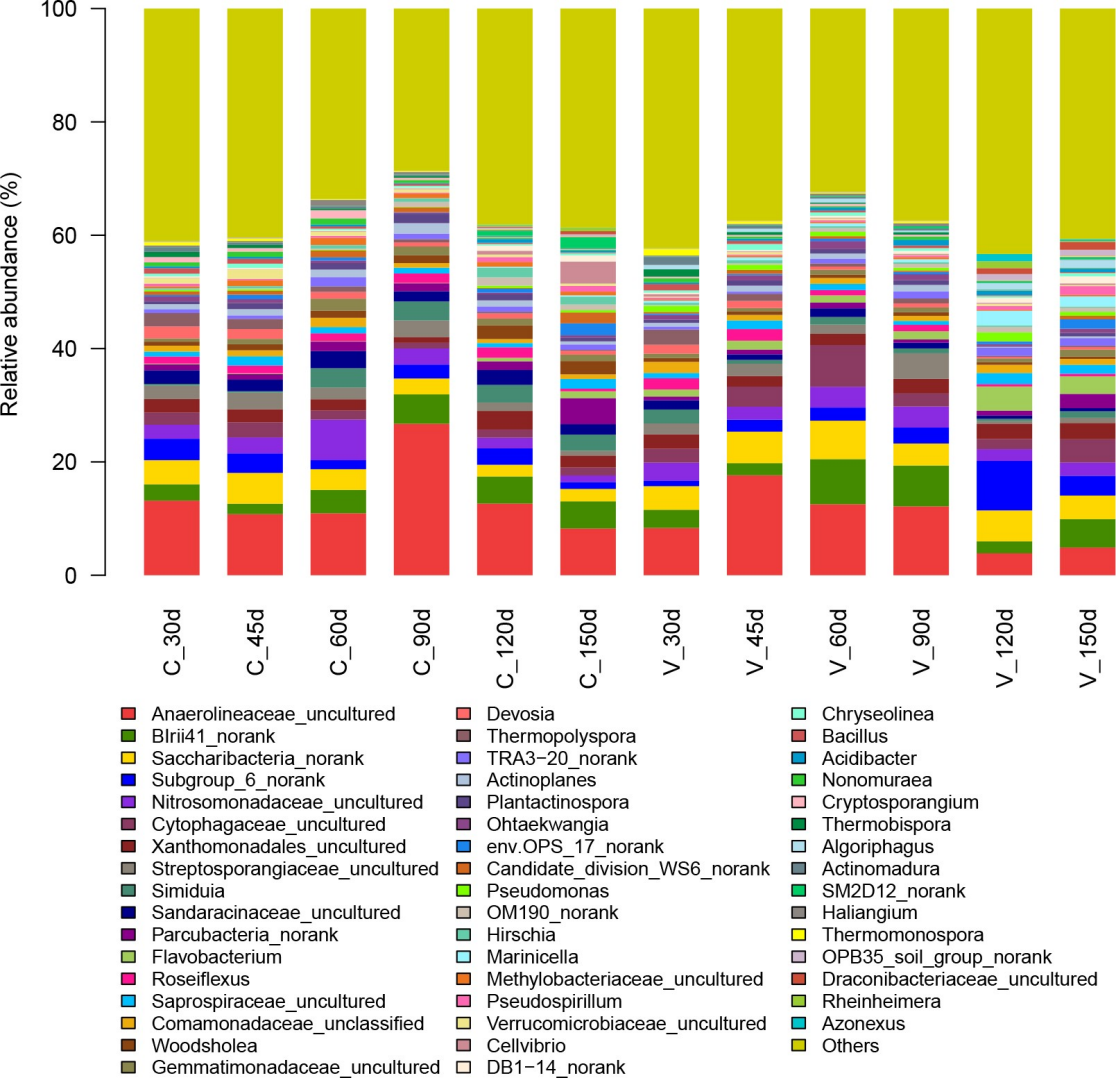
The relative abundance of the bacterial genera from all aerobic compost and vermicompost samples.

As noted earlier, *Proteobacteria w*as the most abundant phylum in both aerobic compost and vermicompost; its relative abundance was 35.1–48.1% in aerobic compost and 32.4–43.1% in vermicompost. Genera of *Proteobacteria* detected in the composts included *BIrii41_nor*, *Nitrosomonadaceae_uncultured*, *Xanthomonadales_uncultured*, *Simiduia*, *Sandaracinaceae_uncultured*, *Comamonadaceae_unclassified*, *Woodsholea*, *Devosia*, *TRA3-20_norank*, *Pseudomonas*, *Hirschia*, *Marinicella*, *Methylobacteriaceae_uncultured*, *Pseudospirillum*, *Cellvibrio*, *DB1-14_norank*, *Acidibacter*, *SM2D12_norank*, *Haliangium*, *Rheinheimera*, and *Azonexus* (S1 Table.). In the aerobic compost at 30 d, the dominant genus was *BIrii41_norank* (order Myxococcales) (Fig 3), which accounted for 2.88% of the total reads. At 45 d to 60 d in the aerobic compost, *Nitrosomonadaceae_uncultured* (order Nitrosomonadales) became the dominant bacterial genus (Fig 3), accounting for 2.83% to 7.17% of the total reads. After 60 d of aerobic composting, *BIrii41_norank* was once again the dominant genus, accounting for 4.77–5.19% of the total reads. The relative abundance of the following genera was low: *Xanthomonadales_uncultured* (order Xanthomonadalesord) (1.03–3.29%), *Simiduia* (0.24–3.37%), *Sandaracinaceae_uncultured* (order Myxococcales) (1.79–3.04%), *Comamonadaceae_unclassified* (order Burkholderiales) (0.72–1.65%), *Woodsholea* (order Caulobacterales) (0.72–2.44%), *Devosia* (order Rhizobiales) (0.69–2.11%), *TRA3-20_norank* (order TRA3-20) (0.61–1.64%), *Pseudomonas* (order Pseudomonadales) (0.09–0.43%), *Hirschia* (order Caulobacterales) (0.41–1.66%), *Marinicella* (order Incertae_Sedis) (0.003–0.22%), *Methylobacteriaceae_uncultured* (order Rhizobiales) (0.34–1.26%), *Pseudospirillum* (order Oceanospirillales) (0.18–1.00%), *Cellvibrio* (order Cellvibrionales) (0.06–3.88%), *DB1-14_norank* (order DB1-14) (0.11–1.10%), *Acidibacter* (order Xanthomonadales) (0.17–0.66%), *SM2D12_norank* (order Rickettsiales) (0.03–1.92%), *Haliangium* (order Myxococcales) (0.10–1.07%), *Rheinheimera* (order Chromatiales) (0.004–0.61%), and *Azonexus* (order Rhodocyclales) (0.002–0.10%). In the vermicompost, except for samples at 45 d, the dominant genus in the phylum *Proteobacteria* was *BIrii41_norank*, accounting for 2.11–7.96% of the total reads. At 45 d in the vermicompost, the genus *Nitrosomonadaceae_uncultured* was slightly more abundant (2.28% vs. 2.14%) than the genus *BIrii41_norank* (order Myxococcales).

*Proteobacteria* was previously found to be the most abundant bacterial phylum in aerobic composts or vermicomposts made from olive-mill waste, sewage sludge, coconut leaves, or cow manure [8, 25, 26, 27]. The myxobacteria in this genus (such as *BIrii41_norank*) are widely distributed in nature and were abundant in the aerobic compost and vermicompost of the current study. The secondary metabolites of myxobacteria have antifungal and antiviral properties; they can inhibit eukaryotic RNA and DNA synthesis and protein synthesis and interfere with heavy metal ion transport and other activities [28, 29]. These chemolithotrophic bacteria (genus *Nitrosomonadaceae_uncultured*) can oxidize ammonia to nitrous acid and can therefore be important in reducing nitrogen loss caused by ammonia volatilization during composting. *Comamonadaceae_unclassified* is in the class *Burkholderiales* and is the pathogen of onion rot disease [30]. Later studies found that *Comamonadaceae_unclassified* can help control plant diseases and promote plant growth and bioremediation; *Burkholderia* spp. produce a variety of metabolites with antibacterial activity [31].

In this study, the abundance of *pseudomonads* was low in all aerobic compost samples (0.09–0.43%) and vermicompost samples (0.72–1.60%). *Pseudomonads* can degrade and utilize various complex compounds for growth and reproduction [26]. Therefore, *pseudomonads* might contribute to lignin degradation and TOC reduction in the composting of green waste. Moreover, the ammonia-oxidizing bacteria *Nitrosomonadaceae_uncultured* co-exist with the denitrifying bacteria *Xanthomonadales_uncultured* and *Pseudomonas*, which facilitates the biological nitrogen removal in both of composting by nitrification and denitrification.

*Cellvibrio* spp. produce hydrolytic enzymes that degrade plant cell walls [32]. Some other bacterial genera (such as *Devosia* and *Methylobacteriaceae*_*uncultured*) belong to the order *Rhizobiales* in the phylum *Proteobacteria*; bacteria in this order contribute to nitrogen fixation and can also release plant growth promoting substances and antibiotics [33]. The genus *SM2D12_norank* is in the order *Rickettsiales* and includes plant pathogens. In summary, the *Proteobacteria* detected during aerobic composting and vermicomposting in the current study were diverse and abundant. They are known to be effective at organic matter decomposition.

After *Proteobacteria*, *Chloroflexi* was the next most dominant phylum during both of composting. As the aerobic composting time increased from 30 d to 60 d, the relative abundance of *Chloroflexi* decreased from 19.1% to 10.2%. At 90 d and 120 d, however, the relative abundance of *Chloroflexi* was substantially higher (32.02% and 17.92%, respectively) mainly because of the increase in the relative abundance of the genus *Anaerolineaceae_uncultured* (order Anaerolineales) (26.7% and 12.7%, respectively). *Anaerolineaceae_uncultured* was abundant during aerobic composting (Fig 3), accounting for 8.3–26.7% of the total reads. *Roseiflexus* was another genus detected in the aerobic compost; its relative abundance was low (0.45–1.85%). During vermicomposting, the relative abundance of *Chloroflexi* ranged from 7.4–22.1%, and *Anaerolineaceae_uncultured* was the dominant genus, representing 3.9–17.6% of the total reads of *Chloroflexi*. In a previous report, *Chloroflexi* was more abundant in the casts of earthworms fed with cow manure rather than with pig manure or horse manure [27]; these differences may be attributed to the different microbial species in the feedstock.

The *Chloroflexi* are facultative anaerobic bacteria that are characterized by its can use oxygen to grow well at high temperatures and can also perform photosynthesis under anaerobic phototrophic environment [34]. In this phylum, some strains of genus *Anaerolineaceae_uncultured* are strictly anaerobic and fatty acid oxidizing bacteria and are able to work with methanogens to degrade carbohydrates [35], while *Roseiflexus* was a genus of filamentous anaerobic bacteria and belong to photosynthetic bacterium [36].

The phylum *Actinobacteria* was more abundant in vermicompost samples than in aerobic compost samples. As the vermicomposting process progressed, the relative abundance of *Actinobacteria* first dropped from 20.7% to 7.6% but then abruptly increased at 90 d to 18.9%; the increase was mainly due to the increase in the genus *Streptosporangiaceae_uncultured* (order Streptosporangiales) (4.53%). *Streptosporangiaceae_uncultured* was the dominant genus of *Actinobacteria* (Fig 3), accounting for 5.7–23.9% of the reads in the phylum. In aerobic compost samples, the relative abundance of *Actinobacteria* decreased from 17.0% to 4.9% as composting progressed, and *Streptosporangiaceae_uncultured* was the most abundant genus, accounting for 13.9–22.9% of the reads. The *Actinobacteria* include spore-forming bacteria (such as those in the following genera detected in this study: *Thermopolyspora*, *Plantactinospora*, *Nonomuraea*, *Thermobispora*, *Actinomadura*, and *Thermomonospora*); because they are spore-forming, they can survive the thermophilic phase of composting [37]. Most *Actinobacteria* found in this study were related to *Streptosporangiaceae_uncultured*. The *Streptosporangiales* can use green waste as a carbon source and have a multi-branched vegetative mycelium that can penetrate lignocellulose; these bacteria secrete a series of extracellular enzymes such as cellulase and hemicellulase [38]. Lignocellulosic degradation by *Actinobacteria* may therefore be crucial for compost maturation. Bacteria in the order *Streptosporangiales* and in other genera in *Actinobacteria* (such as *Streptomyces*) can produce a large number of antibiotics [39] and may suppress several plant pathogens. In the previous study on the vermicomposting of coconut leaf, an imputed metagenomics approach was used to predict that on the 75th day, secondary metabolites were involved in the antibiotic (streptomycin, novobiocin, penicillin, neomycin, and butirosin) biosynthesis pathways, and the existence of antibiotic production pathways coincided with the highly abundance of some actinomycetes (such as Streptomyces spp) [26]. As a result, a high abundance of *Actinobacteria* in the final compost product may be considered desirable.

The phylum *Bacteroidetes* was also detected and was more abundant in vermicompost than in aerobic compost. The relative abundance of *Bacteroidetes* in vermicompost samples increased from 9.2% at 30 d to 16.0% at 60 d, but then decreased to 9.2% at 90 d; the latter decrease was mainly due to the decrease in the abundance of the genus *Cytophagaceae_uncultured* (order Cytophagales) to 2.30%. As vermicomposting continued beyond 90 d, the relative abundance of *Bacteroidetes* increased from 9.1% to 20.3%. *Bacteroidetes* was more abundant in vermicompost than in aerobic compost, which was consistent with previous reports [6, 37]. The Gram-negative *Bacteroidetes* are the group of anaerobic bacteria whose main role in the fermentation system is to break down macromolecules (such as proteins, starches, cellulose and fibrous substances) [34]. Its relative abundance was greater in vermicompost probably because the earthworm intestine provides a suitable environment for the growth of *Bacteroidetes*. The decrease in abundance at 90 d could be attributed to the reduced quantities of readily utilizable proteins and carbohydrates. Moreover, a recent study also showed that vermicomposting at 75 d involved the biosynthesis of secondary metabolites with plant growth promotion properties [26].

In vermicompost samples, 11.9–45.9% of the *Bacteroidetes* sequences were assigned to the genus *Cytophagaceae_uncultured* (order Cytophagales), which are cellulolytic [40]. The relative abundance of *Bacteroidetes* in aerobic compost samples increased with composting time from 30 d to 45 d and from 90 d to 150 d. The relative abundance of *Cytophagaceae_uncultured* was lower in aerobic compost (1.00–2.66%) than in vermicompost. Other genera in the *Bacteroidetes* that could degrade lignocellulose include *Flavobacterium*, *Saprospiraceae_uncultured* (order Sphingobacteriales), *Ohtaekwangia* (order Cytophagales), *env*.*OPS_17_norank* (order Sphingobacteriales), *Chryseolinea* (order Cytophagales), and *Algoriphagus* (order Cytophagales); Previous studies have reported that it is possible to isolate and culture *Flavobacterium*, *Cytophaga* and *Sphingomonas* from sludges rich in alkanes, aromatic hydrocarbons and polycyclic aromatic hydrocarbons [41], and that some strains in *Sphingobacteriales* may degrade aromatic compounds[42], while some strains of *Cytophagales* may degradation of lignin [26]. Therefore, it is speculated that members of *Bacteroidetes* may play an important role in the degradation and utilization of lignin with aromatic nucleus structure in woody plants.

*Acidobacteria* and *Planctomycetes* were also present in both the aerobic compost and vermicompost, and higher relative abundance was observed in aerobic compost samples than that in vermicompost samples. In aerobic compost samples, 36.75–58.70% and 6.27–46.61% sequences in *Acidobacteria* and *Planctomycetes* were assigned to the genus *Subgroup_6_norank* (order Subgroup) and *OM190_norank* (class OM190), respectively. *Saccharibacteria* were other detected bacterial phyla, and more than 99.99% of the sequences at the phylum level belonged to genus of unclassified *Saccharibacteria* for the samples with whatever aerobic compost or vermicompost. Phyla with relative abundances greater than 1% also included *Firmicutes*, *Verrucomicrobia*, *Parcubacteria*, *Gemmatimonadetes*, *Candidate_division_WS6*, and *Spirochaetae*, and these were detected in both of compost. According to a previous study, the most abundant bacterial phylum in casts of the earthworm *Eisenia andrei* feeding on pig manure was *Firmicutes* [27]. These differences in community composition might be related to differences in the raw materials that were composted [43]. Our findings that the bacterial community structure differs in vermicompost vs. aerobic compost are consistent with previous reports [37, 44].

**Fig 4:**
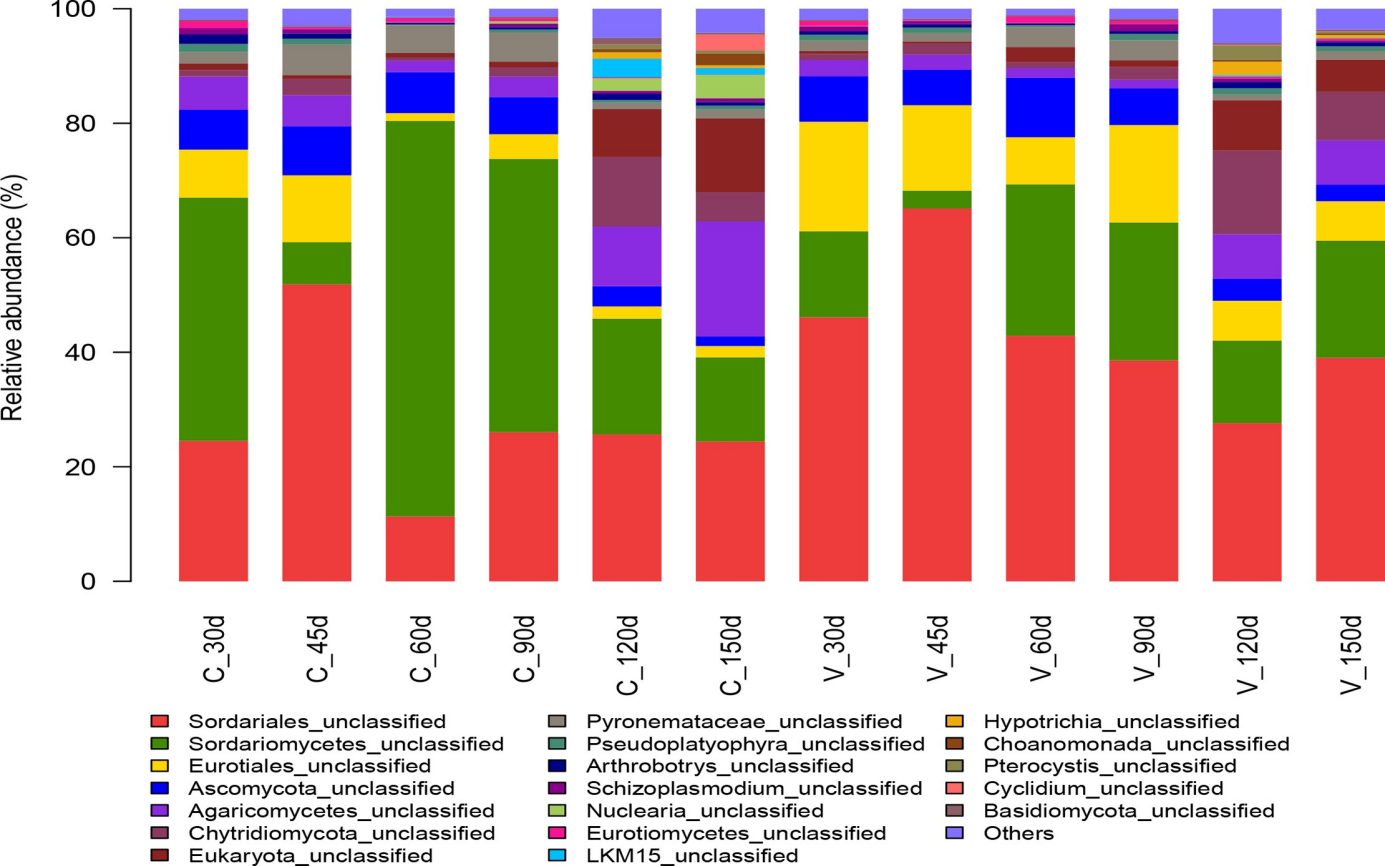
The relative abundance of the fungal and other genera from all aerobic compost and vermicompost samples.

The most abundant fungal phylum in both composts was *Ascomycota*, and its relative abundance was higher in vermicompost samples than in aerobic compost samples. The following classes of *Ascomycota* were detected in all compost samples: Sordariomycetes, Eurotiomycetes, Pezizomycetes, and Orbiliomycetes (Fig 4). In aerobic compost, the relative abundance of *Ascomycota* was 91.5–95.9% in the mesophilic phase (60–90 d), 87.3–88.3% in the thermophilic phase (30–45 d), and 45.5–54.5% at maturity (120–150 d). During aerobic composting, the major class was Sordariomycetes, which accounted for 39.2–80.6% of the total reads, followed by Eurotiomycetes (2.08–12.06%), a class of unclassified Ascomycota (1.68–8.58%), Pezizomycetes (1.42–5.98%), and Orbiliomycetes (0.26–1.68%). Fungal community composition differed in aerobic compost and vermicompost samples. In vermicompost samples, the relative abuandance of phylum *Ascomycota* was 91.6–93.6% in the mesophilic phase (30–90 d) and 55.8–72.3% at maturity (120–150 d), and Sordariomycetes was the prevalent class; its relative abundance ranged from 42.1 to 69.5%. Meanwhile, the abundance of class Eurotiomycetes decreased from 20.0% to 7.2% as vermicomposting time increased. Wang et al. [45] reported that fungal communities significantly changed during aerobic composting of cow dung, but that *Ascomycota* was dominant at all stages of composting. Based on the detection and identification of culturable fungi, Anastasi et al. [12] found that the species richness and abundance of *Ascomycota* were higher in vermicompost than in aerobic compost, mainly due to *Corynascus sepedonium* (class Pezizomycetes), *Eurotium chevalieri* (class Pezizomycetes), and *Talaromyces flavus var*. *flavus* (class Pezizomycetes). Many fungi are thermotolerant or thermophilic, and Wang et al. [45] found that substrate utilization by the fungal community in aerobic compost was highest in the high temperature and maturity phases. Thermophilic fungi can produce thermo-stable enzymes that degrade heterocyclic compound [46]. Anastasi et al. [12] isolated 66 species of fungi from aerobic compost and vermicompost that were thermotolerant and that could degrade various organic wastes. Many fungi are known to use lignocellulosic polymers as a carbon source and are therefore expected to reduce the lignocellulosic content in the aerobic composting and vermicomposting of green waste [47].

The second most abundant fungal phylum in the compost samples was *Basidiomycota*. In the aerobic compost, the relative abundance of *Basidiomycota* decreased from 6.24% to 2.17% from 30–60 d, and then increased from 3.7% to 20.6% from 90–150 d. Most of the sequences were assigned to Agaricomycetes (90.1–99.8%) and a class of unclassified Basidiomycota (0.09–9.79%). In the vermicompost, the relative abundance of *Basidiomycota* decreased from 3.04% to 1.56% at the mesophilic phase (30–90 d) and then increased to about 8% during the curing stage. Agaricomycetes was the dominant class of *Basidiomycota*, accounting for 95.8–99.8% of the total reads. This result was consistent with Anastasi et al. [12], who reported that the *Ascomycetes* was more abundant and diverse than the Zygomycota during vermicomposting. This might be due to the preferential ingestion of Zygomycota by earthworms, which could reduce Zygomycota numbers and benefit K-selected fungi in the *Ascomycetes* and *Basidiomycetes* [48]. In a study of the composting of vegetable waste, Huang et al. [6] found that the dominant fungal classes were the Agaricomycetes and Saccharomycetes in the aerobic compost, while vermicompost was dominated by Sordariomycetes, followed by Agaricomyces, Pezizomycetes, Eurotiomycetes, Saccharomycetes, and Orbiliomycetes in the vermicompost. Most Agaricomycetes are white-rot fungi with the ability to decompose monocyclic and polycyclic aromatic compounds [6], and are probably important for the decomposition of the complex components of green waste.

The phylum *Chytridiomycota* was detected in both aerobic compost and vermicompost samples, and the relative abundance in vermicompost samples was higher than that in aerobic samples. Over the vermicompost time from 30- to 120-d, the OTUs abundance increased from 1.05% to 14.60% and decreased to 8.49% at 150 d. The change trend of OTU abundance of *Chytridiomycota* in aerobic compost was consistent with vermicompost, indicating that it was not affected by the composting method. More than 99% sequences at the class was assigned to unclassified Chytridiomycota for the samples with aerobic compost or vermicompost.

*Chytridiomycota* was considered to be an ancient class that includes aquatic fungi that have been detected in deep-sea hydrothermal ecosystems [49]. The increased abundance of *Chytridiomycota* at the curing stage (120 d) may be related to its adaptation to salt- and brackish-water environments [49], given the slightly alkaline conditions at the end of the green waste composting (Table 1). In addition, the *Chytridiomycota* are anaerobic and may be favored by the relatively anaerobic environment of the earthworm intestine. Previous studies have shown that *Chytridiomycota* can degradation of plant fodder of mammalian herbivores [50].

*Zygomycota* and *Cryptomycota* were also identified in samples of both aerobic compost and vermicompost. *Zygomycota* was detected at all stages of composting, with low relative abundance in both aerobic compost (0.02–0.75%) and vermicompost (0.01–0.44%). *Cryptomycota* was identified only at the curing stage (120–150 d) and at very low levels; its relative abundance was 0.02–0.05% in aerobic compost and 0.01–0.02% in vermicompost.

As mentioned above, compared to aerobic compost, the vermicomposting of green waste is characterized by 1) Improve the degradation of greening waste and the nutrient content of the final compost products, improving the chemical properties of the finished compost products. 2) The richness and variety of microorganisms in the vermicomposting process is relatively high, which increases the degradation rate of greening wastes. 3) Microbial abundance of bacteria such as Actinomyces and Bacteroidetes and Ascomycota in fungi during vermicomposting is relatively high. 4) Microbes of different compost stages in aerobic compost and vermicompost are of the same genus level with different abundance values. These OTU parameters can be used to characterize potentially useful indicators between the quality of the finished compost and the impact of the production process. There are prospects for improving the composting process and providing corresponding application fields.

## Conclusion

The quality of the compost and the structure of the microbial community in the compost differed when green waste was processed by vermicomposting vs. aerobic composting. Relative to the aerobic compost, the vermicompost had a lower pH, lower lignin and cellulose concentrations (*P*<0.05), a higher EC value, and higher TN and available P concentrations. In both composts, the dominant bacterial phylum was *Proteobacteria*, followed by *Chloroflexi*, *Actinobacteria*, *Bacteroidetes*, *Acidobacteria*, and *Saccharibacteria*, and the major fungal phyla were *Ascomycota*, *Basidiomycota*, *Chytridiomycota*, *Zygomycota*, and *Cryptomycota*. The dominant genera at different stages of composting differed between aerobic compost and vermicompost. During aerobic composting, *BIrii41_norank* (order *Myxococcales*) was the major bacterial genus at 30 d and again after 60 d; *Nitrosomonadaceae_uncultured* (order *Nitrosomonadales*) was the major bacterial genus at 45 d and 60 d; and *Sordariomycetes* was the major fungal class from 30–150 d. During vermicomposting, the dominant bacterial genus was *BIrii41_norank* except at 45 d, and the dominant fungal class throughout the process was *Sordariomycetes*.

## Supporting information

S1 Table The type of the detected and undetected bacterial genus and the phylum level at which the genus was located as well as the percentage of bacterial genus relative to a phylum level respectively during the aerobic composting and vermicomposting of green waste. (XLSX)
